# Descemet Membrane Endothelial Keratoplasty after failed penetrating keratoplasty– case series and review of the literature

**DOI:** 10.1186/s12886-023-03279-4

**Published:** 2024-01-08

**Authors:** Agata Anna Wykrota, Loïc Hamon, Loay Daas, Berthold Seitz

**Affiliations:** Department of Ophthalmology, Saarland University Medical Center (UKS), Kirrberger Straße 100, Building 22, 66421 Homburg/Saar, Germany

**Keywords:** Cornea, Descemet Membrane Endothelial Keratoplasty, Graft failure, Graft undersizing, Sequential keratoplasty

## Abstract

**Background:**

This study aims to evaluate visual outcome, central corneal thickness, and re-bubbling rate in a cohort with undersized sequential Descemet Membrane Endothelial Keratoplasty (DMEK) due to endothelial graft decompensation following primary penetrating keratoplasty (PK).

**Methods:**

All patients who received a sequential DMEK (n = 16) or triple DMEK (n = 2) after failed primary PK between November 2020 and June 2022 were retrospectively evaluated. Analyzed parameters were corrected distance visual acuity (CDVA), central corneal thickness (CCT), re-bubbling rate and graft survival.

**Results:**

18 eyes of 18 patients were included. All patients underwent a DMEK with undersized graft after failed PK(s). Mean time between the last PK and DMEK was 102 ± 82 weeks. Mean follow-up time was 8.9 ± 4.6 months. CDVA increased significantly from 1.12 ± 0.60 logMAR preoperatively to 0.64 ± 0.49 logMAR 6 weeks postoperatively (p = 0.013). Mean CCT decreased significantly from 807 ± 224 μm before to 573 ± 151 μm 6 weeks after DMEK (p = 0.003). Re-bubbling was necessary in eight eyes (44.4%) after a median time of 7 days. The 12-month Kaplan Meier survival was 66.7%.

**Conclusion:**

In case of endothelial graft decompensation without stromal scars after primary PK, a DMEK can be performed for selected patients who had satisfying CDVA before the endothelial decompensation. Prior to DMEK indication, an AS-OCT should routinely be performed to circularly search for posterior steps at the PK graft margin, as well as shortly after DMEK to exclude a detachment of the endothelial graft. All patients should be informed about a higher re-bubbling rate in comparison to primary DMEK.

## Background

In Germany, about one third of all primary keratoplasties are currently performed as penetrating keratoplasties (PK) [1]. Potential causes of graft failure after PK are an endothelial immune reaction [2], chronically dysregulated intraocular pressure, especially in association with buphthalmus [3], transplanted endothelial guttae from the donor cornea [4] or secondary graft failure due to herpetic endotheliitis from the recipient (as reactivation of a pre-existing dormant herpes infection in the host cornea) or from the host (transplanted herpes infection) [5, 6].

Our definition of a *primary graft failure* is a graft with no functional response from the first postoperative day. A *secondary graft failure* is a graft with initial postoperative corneal deswelling, but which decompensates after a certain period of time. Further distinction can be made between graft fatigue (decompensation with no specific cause) and decompensation with causal factors e.g., secondary to an immune reaction, a viral infection, etc. [7]. However, the phrase “graft failure” will be used throughout the manuscript to avoid any confusion.

In cases of endothelial graft failure, surgeons are confronted with the dilemma of replacing the entire graft (new PK) or rationally just replacing the compromised endothelial cell layer (sequential DMEK or DSAEK).

Nowadays, a patient who is satisfied with his penetrating graft before endothelial decompensation, with low astigmatism and without stromal scars, seems to have the optimal prerequisite for a successful sequential DMEK (Descemet Membrane Endothelial Keratoplasty) after a failed primary PK. The surgery can also be combined with phacoemulsification and implantation of an intraocular lens (so-called triple DMEK) if lens surgery is needed. Otherwise, re-keratoplasty as PK with a larger graft (typically excimer laser assisted re-PK 8.5/8.6 mm) is generally performed [8].

Recently, there were many studies published regarding sequential DMEK after a failed primary PK. According to these results, the mean decimal corrected distance visual acuity (CDVA) increased after sequential DMEK [9, 10]. Endothelial cell loss typically ranges from 30 to 60% at 1 year after primary DMEK [9–15]. The re-bubbling rate for sequential DMEK after PK varies between 6.3% and 100% in the literature. The results of 4-year survival of sequential DMEK vs. DS(A)EK after failed PK are comparable (76% vs. 74%) [9]. The immune response rate for re-PK after PK is significantly greater than that for sequential DMEK after PK [2, 16]. Different authors suggest different DMEK graft sizes after a failed PK—undersized [17, 18], equally sized [18, 19] as well as oversized [9, 19] grafts were recommended. Undersized grafts should adhere better in the area of the old PK margin, whereas oversized grafts should cover the previous graft-host interface and show more endothelial cells postoperatively.

The purpose of our study was to evaluate visual outcome, central corneal thickness (CCT), re-bubbling rate and graft survival rate for patients who received an undersized sequential Descemet Membrane Endothelial Keratoplasty (DMEK) due to endothelial graft decompensation following primary penetrating keratoplasty (PK) and compare our results to recently published studies regarding this topic.

## Methods

This monocentric, retrospective study analyzed all eyes who underwent a sequential DMEK after failed primary PK between November 2020 and June 2022 at the Department of Ophthalmology at the Saarland University Medical Center (UKS) in Homburg/Saar in Germany, representing 18 eyes of 18 patients. The local ethics committee of Saarland (Ethikkommision bei der Ärztekammer des Saarlandes) was informed about the study and no ethical approval was required according to the committee. This study was conducted in accordance with the Declaration of Helsinki. Indications for a sequential DMEK were secondary corneal decompensation with or without bullous keratopathy. All patients underwent a complete ophthalmological clinical evaluation including the corrected distance visual acuity (CDVA) [logMAR], intraocular pressure (IOP) measurement with Goldmann applanation tonometry, and anterior and posterior segment examination using a slit lamp (Slit Lamp BX 900®, Haag-Streit, Köniz, Switzerland). Additional parameters included the central corneal thickness (CCT) measured with Scheimpflug corneal tomography (Pentacam® HR, OCULUS, Wetzlar, Germany), endothelial cell density (ECD) using specular microscopy (Endothelium Specular Microscope EM-4000, Tomey Corp., Nagoya, Japan), anterior segment optical coherence tomography (AS-OCT CASIA 2, Tomey Corp, Nagoya, Japan) and central macular thickness (CMT) with macular optical coherence tomography (M-OCT) (Spectralis OCT, Heidelberg Engineering, Heidelberg, Germany).

AS-OCT was routinely performed prior to surgical indication for circular assessment of potential unfavorable posterior steps at the former PK graft margin. A neodymium-doped yttrium aluminium garnet (Nd:YAG) iridotomy at 6 o’clock was routinely performed before DMEK in order to avoid postsurgical IOP dysregulations and associated Urrets-Zavalia syndrome [20]. If the peripheral host cornea was too edematous and thus not transparent, large iridectomy at 6 o’clock was performed intraoperatively using the vitrectomy cutter. To improve intraocular visibility, a large corneal abrasion was performed at the beginning of the DMEK surgery. The Descemet’s membrane of the PK graft was always removed, and a 0.5 mm undersized endothelial graft was used (typically with 7.5 mm DMEK) after an 8.0 mm PK. Descemetorhexis was performed within the original donor-recipient junction to avoid opening the stroma-stroma interface between host and graft. In each case, the DMEK donor was marked with three asymmetric semicircles at the margin according to the technique of Bachmann, Cursiefen and Kruse to determine the graft orientation in the anterior chamber [21]. The removed Descemet’s membrane and endothelial cell layer as well as an aqueous humor aspirate was always evaluated by polymerase chain reaction (PCR) for viruses (HSV, VZV, CME and EBV) by our virology department [5]. The DMEK graft was then centered within the PK graft and 20% sulfur hexafluoride 6 (SF6) gas was routinely used at the end of the surgery to reduce the re-bubbling rate. After 100%-filling for 2 to 3 h, some gas was released (“de-bubbling”), thus achieving approximately 90 to 95% filling, and the patient was recommended to be in supine or alternate side position for several days in order to avoid graft detachment. All patients were intensively informed about the possibility of re-bubbling in case of graft detachment. If epithelial removal was performed, a therapeutic contact lens was applied for 2 weeks with topical unpreserved ofloxacin eye drops for prophylaxis of infectious complications and systemic methylprednisolone (100 mg per day with reduction of 20 mg every 2 days) as well as topical steroids (mostly prednisolone acetate eye drops or alternatively loteprednol etabonate eye drops in eyes with higher eye pressure or steroid response) were applied and slowly tapered postoperatively as prophylaxis for immune reactions and cystoid macular edema [22]. Every patient received systemic methylprednisolone 250 mg intravenously for 3 days postoperatively and then orally tapered every 2 days. This therapeutic regimen is equivalent for patients receiving primary DMEK (without PK). In pseudophakic eyes, we recommended to maintain 1 (or 2 in case of previous immune reaction) steroid drops permanently. In eyes with a history of herpetic disease or unclear endothelial decompensation compatible with herpetic background, ganciclovir eye ointment (5 times a day for 6 weeks, then 2 times a day) and systemic aciclovir (400 mg 5 times a day for 6 weeks, then 2 times daily for at least a year) were prescribed additionally. Donor tissues were collected, cultured, and provided by the LIONS eye bank Saar-Lor-Lux; Trier/Westpfalz, our on-site eye bank at the Saarland University Medical Center in Homburg/Saar [23, 24].

Sequential DMEK was always performed in an inpatient setting in order to monitor the IOP as well as possible graft detachment (clinically and using an anterior chamber OCT) early postoperative. After discharge, we recommended weekly check-ups at a general ophthalmologist as well as in our department at 2 and 6 weeks as well as 6 and 12 months postoperatively. If needed, patients could be referred to our department at any time because of e.g. graft detachment, suspected immune reaction or infections. An AS-OCT was performed at 2- and 6-weeks visits to exclude any graft detachment. CDVA, IOP, CCT and ECD were measured preoperatively and at each follow-up in our department. In addition, the re-bubbling rate and graft failure rate after sequential DMEK following PK was analyzed. According to Wu et al., graft failure was defined as the occurrence of a regraft for any reason or, in the absence of a regraft, a cornea that was cloudy postoperatively and did not clear anymore [25].

A pairwise t-test was performed to compare the pre- and postoperative values. A Kaplan-Meier survival analysis was performed to analyze the re-bubbling rate. A p-value of < 0.05 (two-tailed) was considered statistically significant. All the statistical analysis was performed using SPSS Version 2017 for Windows (SPSS Inc., Chicago, IL).

## Results

Eighteen eyes of 18 patients received a sequential DMEK after a failed primary PK between November 2020 and June 2022 at the Department of Ophthalmology at the Saarland University Medical Center (UKS) in Homburg/Saar in Germany. Baseline characteristic data, including cohort data, indications for the first PK, as well as sequential DMEK, are presented in Table 1.Sequential DMEK was performed after an average of 102 ± 82 (median 105) weeks after the last PK. Two eyes were still phakic at the time of the sequential DMEK surgery and underwent a triple DMEK, whereas the rest of eyes had already had a previous cataract surgery and underwent a classical DMEK. Follow-ups were performed after 2 and 6 weeks and after 8.9 ± 4.6 months (then referred to as “last follow-up”).

**Table 1:** Baseline characteristics data

Number of patients	Total 18
	Male 9
	Female 9
Average age	64 ± 16 years
Number of eyes	18
Right eyes	9
Left eyes	9
Number of primary PK	Mean 1.28 (± 0.6)
1	14 (77.8%)
2	3 (16.7%)
3	1 (5.6%)
Indications for primary PK	
Keratoconus	8 (44.4%)
Corneal decompensation with bullous keratopathy due to Fuchs’ endothelial dystrophy	4 (22.2%)
Pseudophakic bullous keratopathy	3 (16.6%)
Keratoglobus	1 (5.6%)
Granular corneal dystrophy	1 (5.6%)
Iatrogenic keratectasia after Laser in situ keratomileusis (LASIK)	1 (5.6%)
Indications for sequential DMEK (secondary corneal decompensation with or without a bullous keratopathy)	
Graft failure	10 (55.6%)
Graft rejection	3 (16.6%)
Transplanted guttae	3 (16.6%)
After cataract surgery (pseudophakic bullous keratopathy)	1 (5.6%)
As a result of intraocular inflammation	1 (5.6%)
Phakic eye at the time of sequential DMEK	2 (11.1%)
Pseudophakic eye	16 (88.9%)

**Fig. 1:**
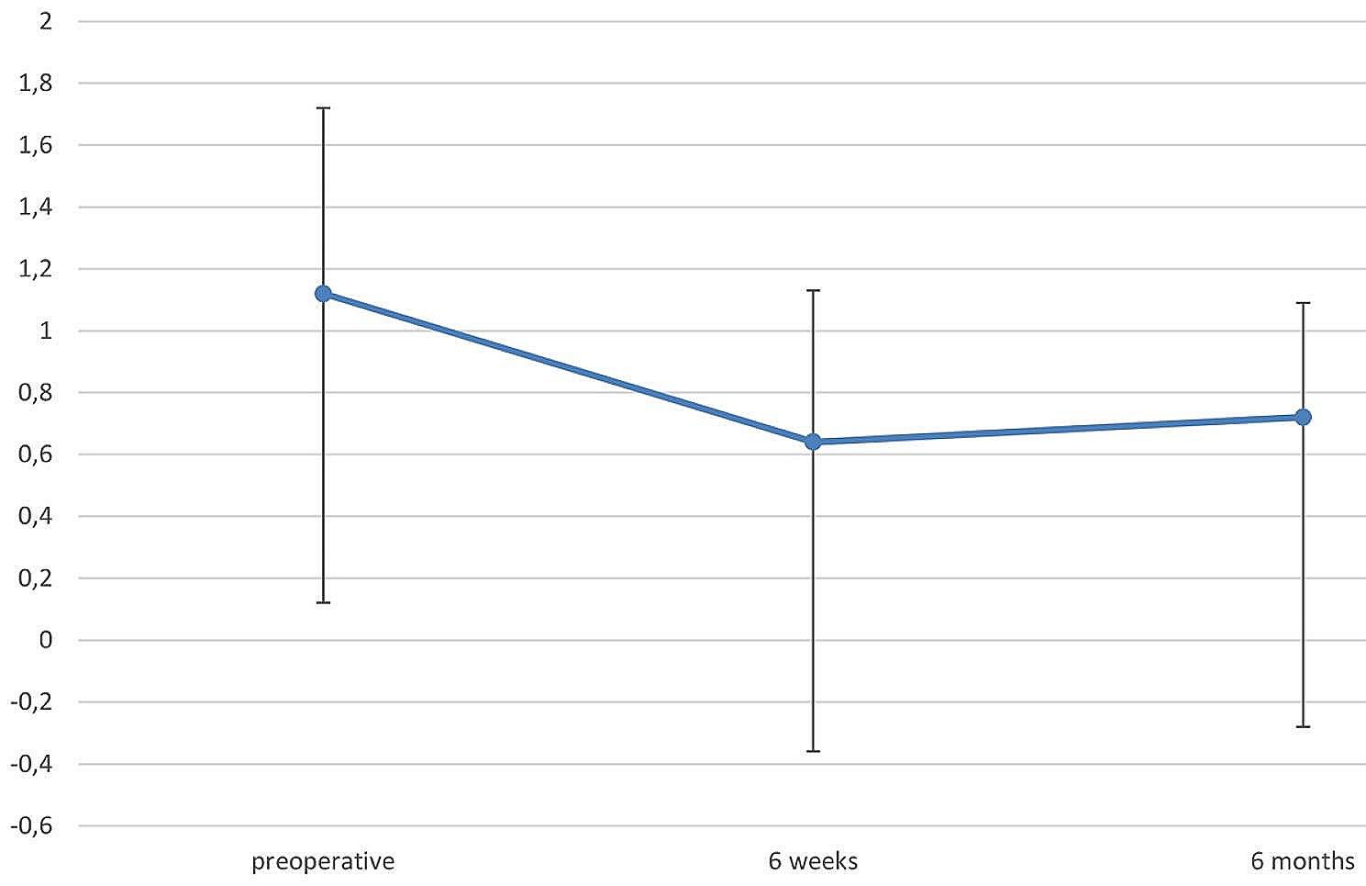
Corrected distance visual acuity (CDVA) in logMAR before and after sequential DMEK as a function of time

The CDVA improved significantly from 1.12 ± 0.60 logMAR preoperatively to 0.64 ± 0.49 logMAR after 6 weeks (p = 0.013) and remained stable with 0.72 ± 0.37 logMAR (p = 0.661) at the last follow-up (Fig. 1).

The mean IOP was 14.2 ± 3.1 (median 14.5) mmHg preoperative and 13.6 ± 5.0 (median 12) after 6 weeks (p = 0.516). It remained stable until the last follow-up with an IOP value of 14.3 ± 3.8 (median 14, p = 0.863). In one case (5.6%) presenting a buphthalmic eye, the IOP raised multiple times to 30 mmHg and remained uncontrollable, leading to secondary corneal decompensation.

**Fig. 2:**
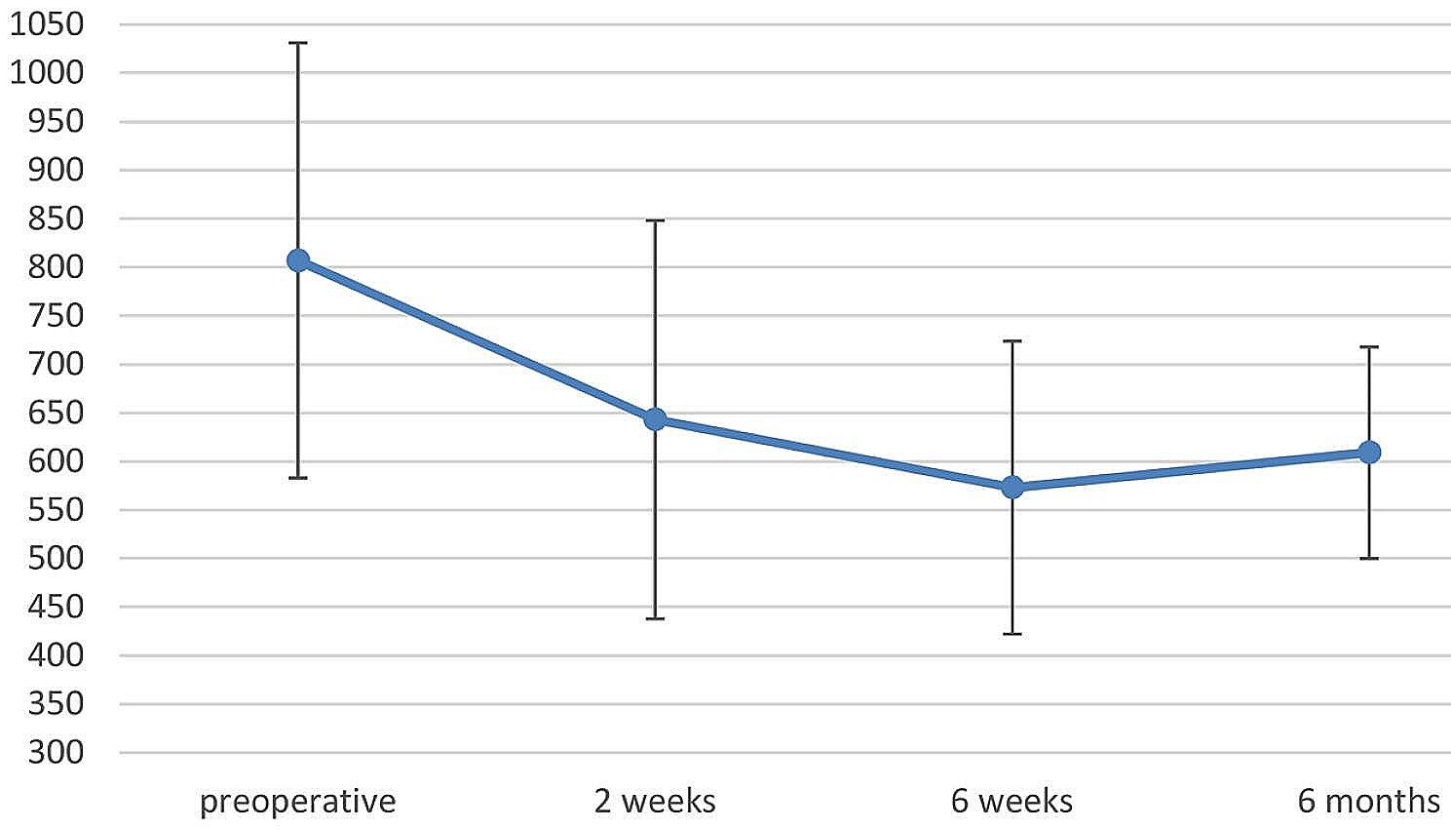
Central corneal thickness (CCT) in µm before and after sequential DMEK as a function of time

The CCT was 807 ± 224 μm preoperatively, corresponding to a decompensated cornea. After DMEK, CCT already decreased to 643 ± 205 μm after 2 weeks (p = 0.091) and to 573 ± 151 6 weeks postoperatively (p = 0.003) (Fig. 2). At the 6 months follow-up (last follow-up), CCT was 609 ± 109 μm on average (p = 0.087).

Donor graft data included the time from patient´s death to recovery of the graft and to the surgery, as well as mean ECD preoperatively. Mean death to recovery and to surgery time was 19.5 h and 24 days respectively. The mean ECD of donor graft preoperatively was 2580 ± 254 cells/mm^2^. The ECD after DMEK was 1010 ± 503 cells/mm^2^ and 1036 ± 657 cells/mm^2^ after 6 weeks and at the last follow-up, respectively.

**Fig. 3:**
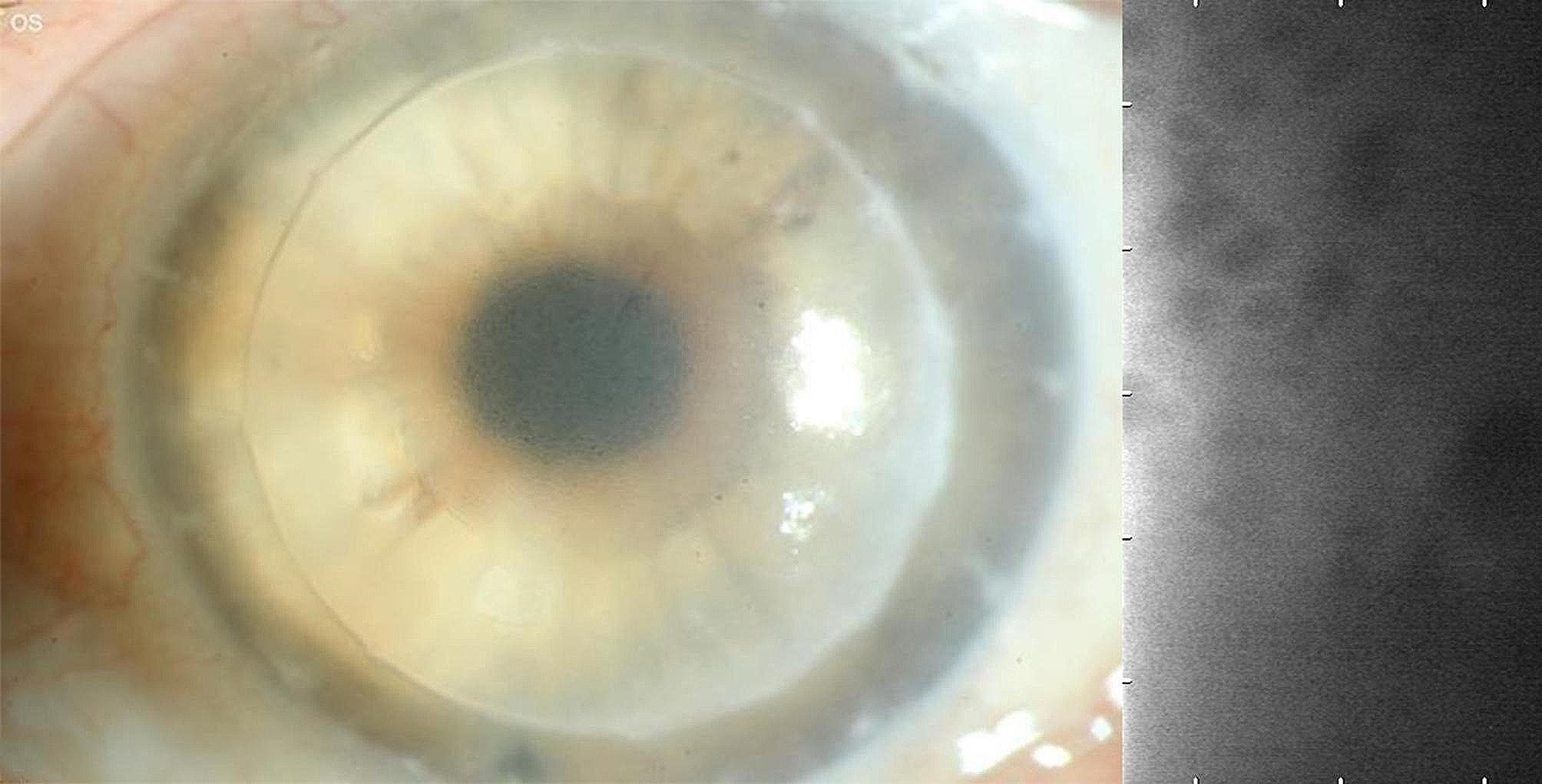
A 78-year-old male patient with secondary corneal decompensation with bullous keratopathy, visual acuity of Snellen decimal 0.2 (logMAR 0.7), IOP 12 mmHg, CCT 870 μm and not measurable ECD due to graft failure

**Fig. 4:**
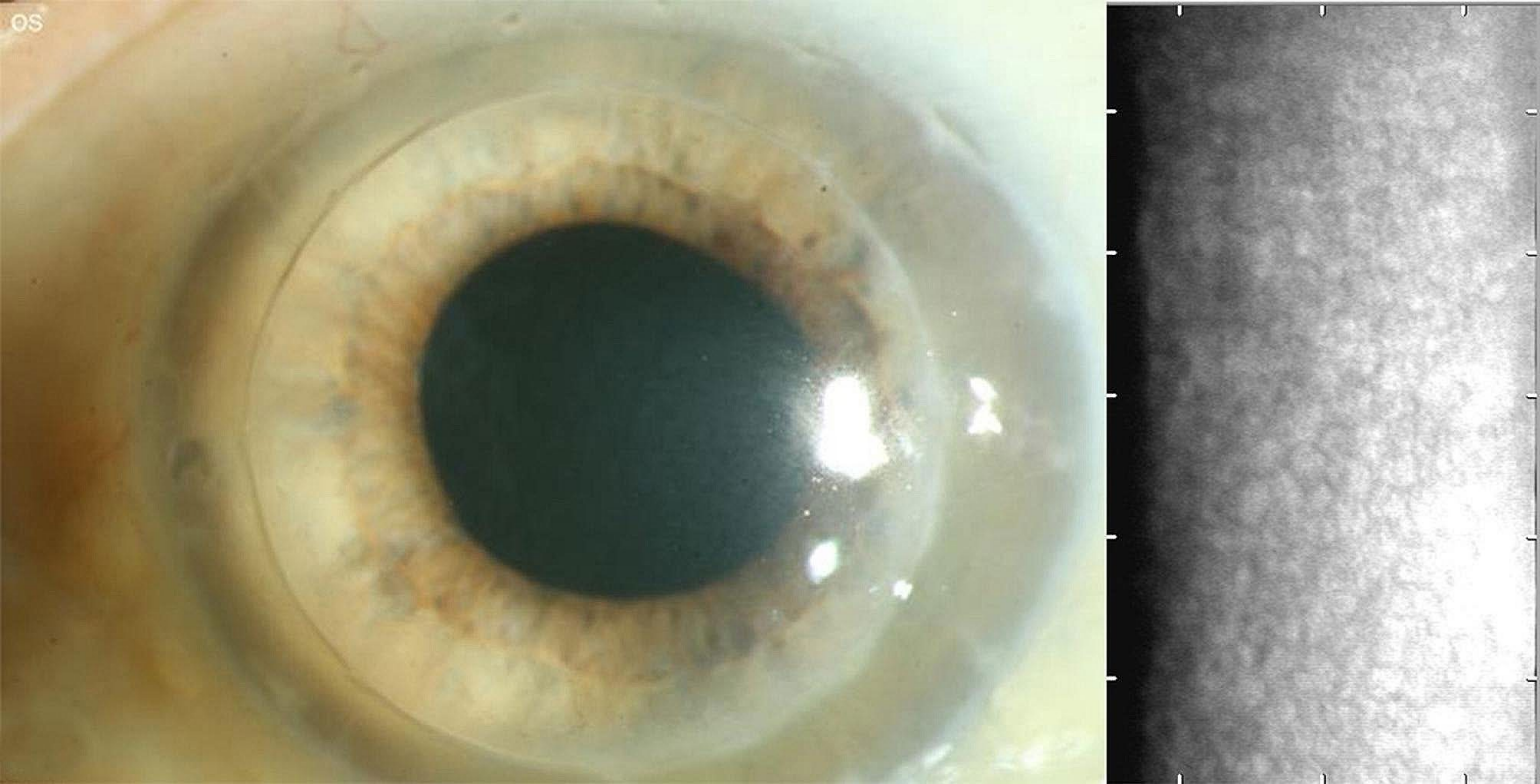
Patient 6 weeks postoperatively with clear sequential DMEK graft, visual acuity of Snellen decimal 0.6 (logMAR 0.2), IOP 17 mmHg, CCT 505 μm and ECD of 1351 cells/mm^2^

We present a case of a 78-year-old male patient (Figs. 3 and 4) who underwent a PK combined with cataract surgery on his left eye in 2012. The indication for primary PK was a Fuchs´ endothelial dystrophy.

**Fig. 5:**
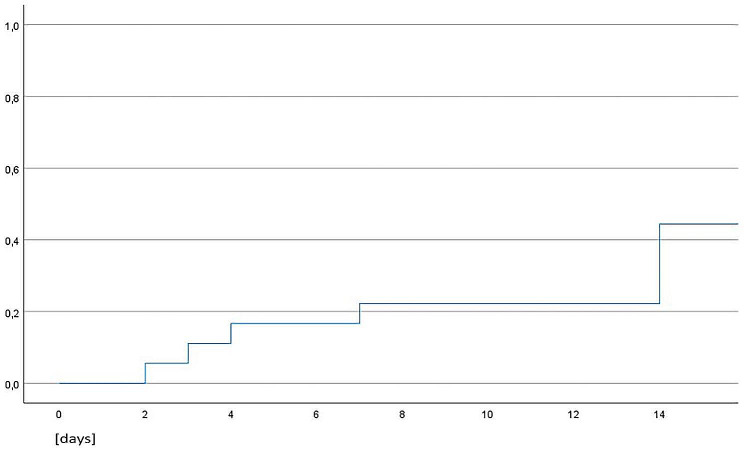
Kaplan-Meier survival analysis of re-bubbling rate after sequential DMEK in days

Re-bubbling was necessary in eight eyes (44.4%, once per each eye) due to graft detachment, with a median re-bubbling time of 7 days after the surgery (Fig. 5). There was no difference in re-bubbling rate between patients who underwent a neodymium-doped yttrium aluminium garnet (Nd:YAG) iridotomy and patients with an intraoperatively performed iridectomy using the vitrectomy cutter. One patient underwent a XEN implantation for glaucoma prior to the sequential DMEK and showed a massive gas outflow already 1 day after DMEK. Despite successful re-bubbling, the patient continued to have a gas outflow through the XEN implant. Nevertheless, no further re-bubbling was needed, as the graft remained attached.

Immunological graft rejection occurred in five cases (27.8%), after 3 months (n = 1), 6 months (n = 2), 8 months (n = 1) and 12 months (n = 1). A re-DMEK was performed again in five cases, whereas one eye underwent a sequential PK. Five eyes (27.8%) presented with a postoperative macular edema after 2 (n = 1), 4 (n = 1), and 6 weeks (n = 3), and were treated with nonsteroidal anti-inflammatory eye drops topically and systemic acetazolamide, showing complete edema resorption in all 5 cases.

**Fig. 6:**
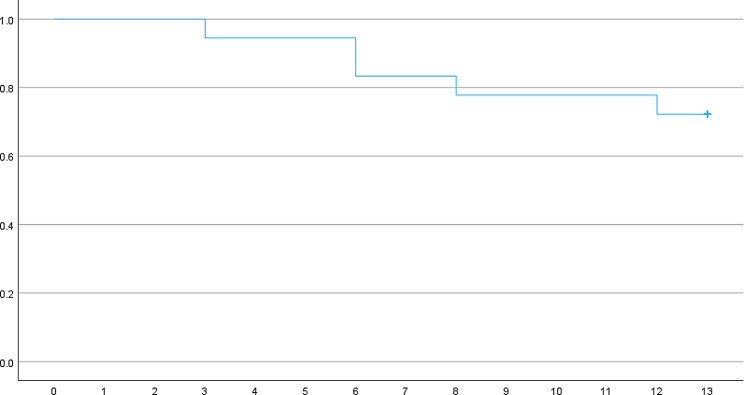
Kaplan Meier survival analysis of 18 DMEK grafts as a function of time in months

Out of 18 eyes, which underwent a sequential DMEK after failed primary PK, 12 (66.7%) survived and no transplantation was needed at the time of the last follow-up (Fig. 6).

## Discussion

For more than a decade, DMEK has been considered the method of first choice in Germany for corneal endothelial decompensation without stromal scars [21]. The main indication in developed countries remains the Fuchs endothelial dystrophy by far. In addition, DMEK is indicated in pseudophakic bullous keratopathy, pseudoexfoliation (PEX) keratopathy, endothelial decompensation after herpes simplex virus (HSV) endotheliitis [26] or buphthalmus, presenting typical ruptures of the Descemet’s membrane and endothelium (Haab striae) [27]. Basically, DMEK is a priori not suitable in cases of long-standing corneal decompensation with stromal scars and in cases of pre-descemetal stromal scars (e.g., after interstitial keratitis). Finally, yet importantly, DMEK is also a potential option for the management of secondary graft failure after PK with/without bullous keratopathy but without stromal scars [7]. This condition is classically due to graft failure (e.g. in cases of transplanted guttae) [4, 28], after endothelial immune responses, and implantation of glaucoma drainage devices [9]. When DMEK is successfully performed, patients benefit from much faster visual rehabilitation compared to PK. In addition, DMEK showed a significantly lower immune response rate compared to PK [16]. Moreover, DMEK surgery avoids the risk of suture complications (e.g. infections or loose sutures) [29] and expulsive bleeding is extremely unlikely with DMEK.

Some authors already investigated the possibility and outcomes of sequential lamellar grafts after failed PK. Wu et al. recently published a literature review on indications and outcomes of Descemet’s Stripping (Automated) Endothelial Keratoplasty (DS(A)EK) and DMEK for the treatment of failed primary PK. A total of 25 studies involving 989 eyes of 970 patients were included, of which 243 eyes underwent DMEK surgery. The review showed an improvement of visual acuity, as well as mean graft survival rate of 76% at 4 years post-DMEK operation in DMEK group [25].

A sequential DMEK after PK should be performed instead of a re-PK if a toric IOL has already been implanted in the eye to compensate for high astigmatism, otherwise the axis of the toric IOL will no longer correspond to the keratometry of the new PK graft. However, toric IOL are often hydrophilic, which could lead to superficial central calcification of the IOL optics after DMEK using gas. Therefore, it is recommended to use 20% SF6 gas tamponade [19]. If an optically disruptive cataract exists at the time of graft failure, triple DMEK should be performed to avoid sequential phacoemulsification with potential re-impairment of the grafted endothelium. In case of associated regular astigmatism, a triple DMEK with a toric IOL remains a valuable option [30].

Contraindications to DMEK after PK include stromal scars on the graft or high and/or irregular astigmatism—especially if the patient cannot tolerate rigid gas-permeable contact lenses. As already mentioned, an AS-OCT is mandatory prior to indication to rule out circular posterior steps in the donor-recipient junction. Such steps predispose to peripheral graft lift-off after sequential DMEK, and a re-PK should rather be considered.

### Graft diameter

The diameter of the sequential DMEK graft was evaluated differently in the literature. In 2019, Pasari et al. reported a re-bubbling rate of 53% with 0.5 mm oversized grafts [9]. In 2018, Pierné et al. reported a re-bubbling rate of 50% with undersized grafts of 0.25 mm [17]. In our study and more generally in our department, we used a 0.5 mm undersized DMEK graft and showed 44.4% of graft detachment that needed re-bubbling, which remains consistent with the previous studies and seems to confirm a slightly lower rate of re-bubbling in case of undersized graft. Heinzelmann et al. postulated that an equally sized or undersized DMEK graft results in a lower re-bubbling rate. A possible explanation for this would be that oversized DMEK grafts adhere less well in the area of the old PK margin with an irregular stromal surface or posterior steps [18]. In contrast, Ang et al. recommended an equally sized or an oversized DMEK graft to cover the previous graft-host interface and have more endothelial cells postoperatively [19].

### Descemetorhexis

The need for descemetorhexis in the PK graft is also controversial in the literature. Alió del Bario et al., Ang et al., and Nottage et al. did not perform descemetorhexis in all patients [11, 12, 19], performing DMEK without prior descemetorhexis as long as the host Descemet’s membrane was intact and has no abnormalities such as guttae, scarring, or retrocorneal precipitates. Pierné et al. reported more frequent graft detachment in cases of incomplete descemetorhexis [17]. Furthermore, a complete descemetorhexis avoids the formation of interface scarring [13]. In 2018, Einan-Lifshitz et al. postulated that femtosecond laser-assisted DMEK may have some advantages after PK [10]. The authors used the femtosecond laser only for the creation of a circular transection of the Descemet’s membrane of the opacified PK graft just inside the circular donor-recipient junction to leave it as unaffected as possible during the subsequent manual descemetorhexis.

In our study, we performed a descemetorhexis and routinely removed Descemet’s membrane of the PK graft manually using continuous air filling of the anterior chamber via anterior chamber maintainer. However, this descemetorhexis seems to be more difficult to succeed after PK (multifocal lamellar clefts, adherence in the donor-recipient interface) than in naive corneas [7, 21].

### Re-bubbling rate

The re-bubbling rate for sequential DMEK after PK varies between 6.3% and 100% in the literature. The “stringency” of the indication and the timing of re-bubbling play a significant role concerning this important divergence. Graft detachment in sequential DMEK after PK appears to be delayed and potentially more extensive than after primary DMEK. Thus, after initial complete adhesion immediately after surgery, up to half of the graft area may be detached again after 1 to 4 weeks in cases of graft detachment [17].

Postoperative ocular hypotony, previous filtering surgery, or a history of ocular hypotony are discussed as causes of graft detachment, but the DMEK graft diameter is one of the most important parameters. A large DMEK graft diameter leads to graft detachment more frequently. According to Lavy et al., the following points should be considered before surgery: (1) do not oversize the DMEK graft too much, (2) educate patients about the possible need for multiple re-bubblings, and (3) avoid postoperative hypotension [13].

Our results show a re-bubbling rate of 44.4%, including one eye after a XEN implantation, which needed re-bubbling already 1 day after DMEK.

### Visual outcomes

Pasari et al. reported a mean decimal CDVA of 0.63 (20/30, logMAR 0.2) at 6 months after sequential DMEK following failed PK [9]. Einan-Lifshitz et al. demonstrated that CDVA continues to increase up to 6 months after DMEK [10]. As expected, the postoperative CDVA seems to be limited by worse preoperative visual acuity [17].

In our study, mean CDVA significantly improved from 1.12 ± 0.60 (Snellen decimal 0.08, 20/250) to 0.64 ± 0.49 (0.25, 20/80) logMAR between before and 6 weeks after sequential DMEK, and stayed stable at 0.72 ± 0.37 (0.2, 20/100) logMAR after 6 months (last follow-up).

### Endothelial cell loss

According to the literature, endothelial cell loss typically ranges from 30 to 60% at 1 year after primary DMEK [9–15]. The postoperative endothelial cell count ranged around 1000 cells/mm^2^ in the present study. Using so-called “precut tissue”, storage of a precut rolled graft in dextran for several days results in endothelial cell loss and worse postoperative outcomes in terms of visual acuity and corneal thickness [31, 32] and thus should be avoided for DMEK after PK.

### DMEK graft failure after PK

In the current literature, the results of 4-year survival of sequential DMEK vs. DS(A)EK after failed PK are comparable (76% vs. 74%) [9]. In the study of Pasari et al., previous glaucoma surgery was the only significant risk factor for failure of DMEK after PK [9]. Particularly, past glaucoma shunt surgery significantly affects the composition and flow behavior of the anterior chamber aqueous humor. This leads to acceleration of renewed endothelial decompensation.

In our study, one eye presented a status post XEN implantation and, therefore, an almost immediate gas outflow from the anterior chamber through the XEN implant. We performed a re-bubbling in this case, which however had the same outcome with reiterated gas outflow. Nevertheless, the DMEK graft stayed attached and no further interventions were needed.

According to studies, the immune response rate for re-PK after PK is significantly greater than that for sequential DMEK after PK [2, 16]. However, we documented five cases (36%) of graft rejection after 3 months (n = 1), 6 months (n = 2), 8 months (n = 1) and 12 months (n = 1). Therefore, the rate of graft rejection after sequential DMEK on primary PK seems to be far superior to the rejection rate after primary DMEK. A possible explanation could be that eyes which have undergone many interventions are more susceptible to graft rejection. Another reason might be the previous, in some cases early, PK failure. For this reason, we recommend the durable use of steroid eye drops once or twice a day for those eyes.

### Limitations

Study limitations include the retrospective aspect of this study, a relatively small cohort of 18 patients, short follow-up period, as well as lack of a control group with grafts of the same size or oversized to compare results after different surgical DMEK techniques. Therefore, further studies on this topic are needed.

## Conclusion

In conclusion, in case of endothelial graft decompensation after PK without stromal scars, a DMEK is a valuable option for selected patients who presented satisfying visual acuity with their PK graft before decompensation and/or are contact lens tolerant. Prior to indication, an AS-OCT should be routinely performed to circularly search for posterior steps at the PK graft margin. In cases of high/irregular astigmatism, DMEK should not be considered, and a well-centered excimer laser assisted re-PK with larger graft diameter (typically 8.5/8.6 mm) to simultaneously treat edema, scarring, and irregular curvature should rather be preferred.

## Data Availability

The dataset supporting the conclusions of this article is available on request to the corresponding author.

## Declarations

All methods were carried out in accordance with relevant guidelines and regulations.
